# Genotoxicity of Aluminum and Aluminum Oxide Nanomaterials in Rats Following Oral Exposure

**DOI:** 10.3390/nano10020305

**Published:** 2020-02-11

**Authors:** Pégah Jalili, Sylvie Huet, Rachelle Lanceleur, Gérard Jarry, Ludovic Le Hegarat, Fabrice Nesslany, Kevin Hogeveen, Valérie Fessard

**Affiliations:** 1 Unité de Toxicologie des Contaminants, Agence Nationale de Sécurité Sanitaire (ANSES), 10 B rue Claude Bourgelat, 35306 Fougères, Francesylvie.huet@anses.fr (S.H.); rachelle.lanceleur@anses.fr (R.L.); gerard.jarry@anses.fr (G.J.); ludovic.lehegarat@anses.fr (L.L.H.); kevin.hogeveen@anses.fr (K.H.); 2 Institut Pasteur de Lille, Laboratoire de toxicologie génétique, 1 Rue du Professeur Calmette, 59019 Lille CEDEX, France; fabrice.nesslany@pasteur-lille.fr

**Keywords:** genotoxicity, comet assay, micronucleus assay, nanomaterial, aluminum, oral route, gut, liver

## Abstract

Due to several gaps remaining in the toxicological evaluation of nanomaterials (NMs), consumers and public health agencies have shown increasing concern for human health protection. In addition to aluminum (Al) microparticles, Al-containing nanomaterials (Al NMs) have been applied by food industry as additives and contact materials. Due to the limited amount of literature on the toxicity of Al NMs, this study aimed to evaluate the in vivo genotoxic potential of Al^0^ and Al_2_O_3_ NMs after acute oral exposure. Male Sprague-Dawley rats were administered three successive gavages at 6, 12.5 and 25 mg/kg bw. A comparison with AlCl_3_ was done in order to assess the potential effect of dissolution into Al ions. Both DNA strand breaks and oxidative DNA damage were investigated in six organs/tissues (duodenum, liver, kidney, spleen, blood and bone marrow) with the alkaline and the Fpg-modified comet assays. Concomitantly, chromosomal damage was investigated in bone marrow and colon with the micronucleus assay. The comet assay only showed DNA damage with Al_2_O_3_ NMs in bone marrow (BM), while AlCl_3_ induced slight but non-significant oxidative DNA damage in blood. No increase of chromosomal mutations was observed after treatment with the two Al MNs either in the BM or in the colons of rats.

## 1. Introduction

Micro-and nanoscale forms of aluminum (Al) have a great lightness and mechanical resistance, and strong oxidizing power [1]. Due to these unique properties, Al microparticles and Al-containing nanomaterials (Al NMs) have been applied by industry, including in food products [2]. Indeed, they are used as food additives (firming agents, anticaking agents, neutralizing agents, emulsifying agents or texturizers) and in food contact materials, such as cooking tools and food packaging [1,2,3,4,5]. In addition, Al-containing particles are largely used in waste water treatment [6], and in drug vehicles, dental products and other hygiene products, such as toothpaste [1,7,8,9].

According to the European Food Safety Authority (EFSA) and the Food and Agriculture Organization/World Health of the United Nations (FAO/WHO), provisional tolerable weekly intakes of 1 and 2.3 mg Al/kg/week have been established respectively [1,10]. However, these organizations have raised the fact that this amount can be exceeded to a large extent by some populations, particularly children. As recurrent exposure to micro- and nanoscale Al particles for the general population occurs through foodstuffs, hazard assessment through ingestion should be addressed. Nevertheless, several gaps remain in the toxicological evaluation of Al NMs following oral exposure, which may raise the concern already outlined concerning Al by consumers and public health agencies.

Few in vivo studies are available on the oral toxicity and genotoxicity of Al NMs, most of them focusing on Al_2_O_3_ NMs. These NMs were shown to accumulate in several organs, including the liver following oral exposure [11,12,13]. Rodents treated orally with 500 to 2000 mg/kg bw of Al_2_O_3_ NMs showed deleterious effects in the liver and kidney, and genotoxicity in bone marrow (BM) and oxidative stress in liver [11,14]. According to the ECHA’s safety assessment, the data available are inconclusive as to whether or not Al_2_O_3_ NMs present a genotoxic potential [15]. In contrast, no data on the genotoxicity of Al^0^ NMs have been published so far, either in vitro or in vivo.

In vitro studies reported that Al_2_O_3_ NMs can induce genotoxicity in a variety of mammalian cell lines, including primary human fibroblasts [16], hepatic HepG2 cells [17], human peripheral lymphocytes [18] and Chinese hamster ovary cells (CHO-K1) [19]. These genotoxic effects were found to be associated with oxidative damage [17] although other studies reported no association [16,19].

In addition, there is a lack of information concerning the mechanisms involved, and whether the release of the ionic compound can be the cause of genotoxicity. Indeed, the metal salt AlCl_3_ has been shown to induce DNA damage in vitro in human peripheral blood lymphocytes, including increases in micronuclei and chromosomal aberrations and positive results in the comet assay [20,21,22]. In vivo, Paz et al. (2017) reported histopathological lesions in the stomach and the liver, and increased chromosomal damage in BM from 50 mg/kg bw after unique oral exposures to mice [22].

In this study, we aimed to provide new genotoxicity data for Al^0^ and Al_2_O_3_ NMs and to do a comparison with AlCl_3_. For this purpose, we conducted an in vivo study in rats by gavage. After three oral administrations, the alkaline and Fpg-modified comet assays were performed on several organs to detect DNA breaks and the micronucleus assay to detect if any chromosomal damage could be induced in bone marrow and the colon.

## 2. Material and Methods

### 2.1. Chemicals, NMs and Dispersion

AlCl_3_ (hexahydrate, 231-208-1) was purchased from Sigma Aldrich (Saint Louis, MO, USA).

The selected Al^0^ and Al_2_O_3_ NMs were obtained from IoLiTec (Heilbronn, Germany) and were chosen with a similar primary particle size (approximately 20 nm). The NANOGENOTOX protocol was used for NM dispersion [23,24]. Briefly, particle powder was dispersed in a scintillation vial at a concentration of 2.56 mg/mL in 0.05% BSA in ultra-pure water (dispersion stock solution) by sonication in ice for 16 min at 400 W using a Branson ultrasonic sonicator (Branson Ultrasonics, Eemnes, Netherlands) with a 13 mm probe diameter. Several parameters of physicochemical characterization in the dispersion stock solution and those provided by the suppliers are summarized in Table 1. A more detailed description of the physicochemical characterization of the two NMs was published previously [25,26].

### 2.2. Animals and Experimental Design

Male Sprague-Dawley (SD) rats, 8–10 weeks old (around 200 g), were purchased from Janvier (Saint Berthevin, France). Rats were housed in conventional cages and had free access to water and food. Temperature and humidity were kept constant with a light/dark cycle of 12 h/12 h. The animals were treated after at least 5 days of acclimatization. All the experiments were in accordance with the ethical recommendations of the Directive 2010/63/EU of the European Parliament and were validated by the Anses ethical committee (COMETH). Five animals per group were randomly assigned to nine groups including negative and positive controls.

Animals were treated by oral gavage (9.76 mL/kg) at 0, 24 and 45 h. Animals were sacrificed 3 h after the last administration. Al^0^ and Al_2_O_3_ NMs were given at 6, 12.5 and 25 mg/kg bw, and AlCl_3_ was given at 25 mg/kg bw. We chose to give a similar mass of compounds to the animals. Nevertheless, the content of Al per animal differed according to the material administered (Table S1). Ultra-pure water with 0.05% BSA was used as vehicle for the negative control group. The positive control was methyl methane sulfonate (MMS from Acros, Geel, Belgium) at 100 mg/kg for the first two oral administrations and at 80 mg/kg for the third. The experimental design was carried out according to the OECD guideline 489 for the comet assay [27].

### 2.3. Tissue Collection and Sample Preparation

Animals were anesthetized with an intraperitoneal sublethal dose of pentobarbital (60 mg/kg), and the following samples were collected: blood, bone marrow from femur, liver, spleen, kidney, duodenum and colon.

For the comet assay, cells or nuclei were isolated as described in Tarantini et al. (2015) [28]. Briefly, blood was collected directly from the heart before a perfusion step; nuclei of the liver and kidney were mechanically isolated from few small pieces using a Medimachine (BD Biosciences, Le-Pont-de-Claix, France) (5 s in the grinding medium). Spleen cells were harvested by flushing. Sections of duodenum and colon were rinsed with Hank’s balanced salt solution (HBSS) medium and epithelial cells were collected by scraping with a coverslip. The rest of the colon and bone marrow cells collected from the two femurs by aspiration with fetal bovine serum were further prepared for the micronucleus assay as described below.

### 2.4. Alkaline Comet Assay and FpG-Modified Comet Assay

Briefly, after isolation from organs, cells were centrifuged 5 min at 136× *g*, and the alkaline comet assay and the modified comet assay using the bacterial DNA repair enzyme formamidopyrimidine-DNA glycosylase (Fpg) were performed (2 slides per organ and condition, migration 24 min, 0.7 V/cm and 300 mA) as previously described [28]. FpG (5U/slide) favors the detection of oxidized bases by catalyzing excision of oxidized purines, including the major purine oxidation product 8-oxoguanine, into single-strand breaks [29]. For each tissue and condition (with and without FpG), two slides were prepared. Before scoring, slides were coded, stained with propidium iodide (2.5 μg/mL in PBS) and immediately observed with a fluorescence microscope (Leica DMR, Nanterre, France) equipped with a CCD-200E camera. For each slide, 75 nucleoids were scored using the Comet Assay IV software (Perceptive Instruments, Haverhill, UK). The percentage of DNA in the tail (% Tail DNA) was chosen to evaluate DNA damage. The mean of the median tail intensity value of each slide was calculated for each animal, prior to the calculation of the mean value of each group. When DNA damage was too high to score, the cells were counted as hedgehogs [30,31].

### 2.5. Bone Marrow Micronucleus Assay (BMMN)

The BMMN assay was carried out following the general principles of the OECD guideline 474 [32]. Briefly, after isolation and centrifugation for 5 min at 210× *g*, drops of BM cells were spread on a microscope slide and allowed to air dry half a day. After fixation in ethanol 96°, the smears were stained for 3 min with May–Grünwald (MG) reagent, 2 min in MG diluted in Sörensen buffer (50/50V, pH 6.75 ± 0.05), 10 min in 14% Giemsa and 1 min in demineralized water. Duplicate slides were prepared for each animal. At least 2000 polychromatic erythrocytes (PCEs) per slide were examined microscopically to determine the frequency of micronucleated polychromatic erythrocytes (MN-PCEs). For myelotoxicity evaluation, the ratio of PCEs to normochromatic erythrocytes (NCEs) was calculated. Coded slides were analyzed under a bright field microscope and micronuclei were scored by two independent scorers.

### 2.6. Colon Micronucleus Assay

The “swiss roll” technique was performed as previously described [28]. The whole colon was cut longitudinally prior to a wash with HBSS. The tissue was rolled up from the rectum to the caecum with the mucosa inward, fixed in 4% formaldehyde and embedded in paraffin. Sections were deparaffinized and dehydrated twice in toluene followed by successive baths of ethanol 100%, 95% and 70%. After rinsing and staining with Schiff’s reagent (Saint Louis, MO, USA) and fast green, a dehydration step in ethanol 70° and ethanol 96° was performed, and finally, slides were mounted using DPX. Intact colon crypts were chosen, and scoring was done on at least 1000 cells per rat.

### 2.7. Statistical Analysis

For the in vivo comet assay, the results from the five animals were analyzed using a one-way ANOVA. For the bone marrow and colon MN assays, Pearson’s chi square test with Yate’s correction was used. Myelotoxicity and hedgehogs over the vehicle control were analyzed with the Mann– Whitney U test (one tailed). Statistical significance was set at *p* < 0.05.

## 3. Results

### 3.1. Comet Assay

**Figure 1:**
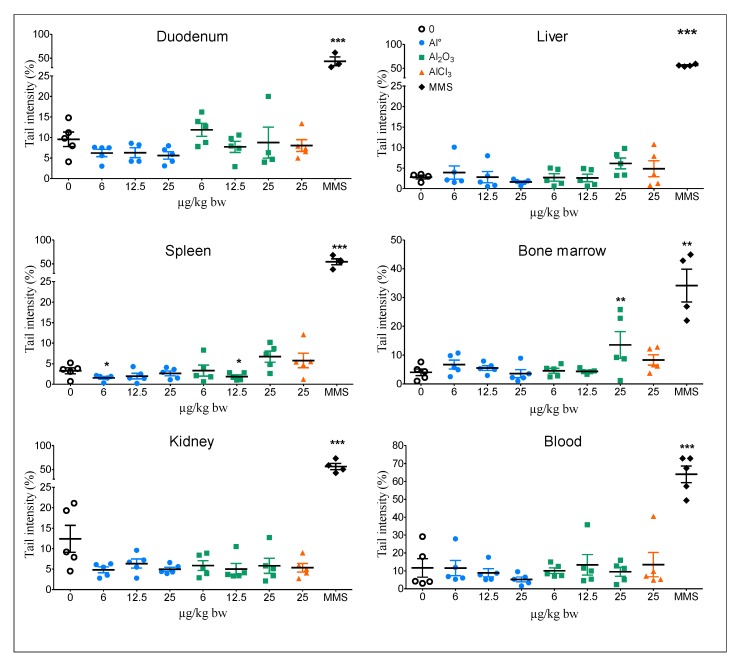
DNA damage in organs/tissues of rats orally exposed to Al^0^ NMs, Al_2_O_3_ NMs or AlCl_3_. A group treated with the vehicle (0, negative control) and a group treated with a genotoxic agent (MMS, positive control) were included. Significant from control at ** *p* < 0.01, *** *p* < 0.001.

The results from the alkaline comet assay after three oral treatments with Al^0^ NMs and Al_2_O_3_ NMs at 6, 12.5 or 25 mg/kg bw/day, and AlCl_3_ at 25 mg/kg bw/day, are shown in Figure 1. Al^0^ NMs did not induce an increase in tail intensity irrespective of the organ or tissue investigated compared to the vehicle control group. Al_2_O_3_ NMs induced a significant increase in tail intensity only in BM at the highest dose (25 mg/kg bw).

AlCl_3_ did not induce increases in the DNA tail intensity irrespective of the organ or tissue investigated compared to the vehicle control group.

**Figure 2:**
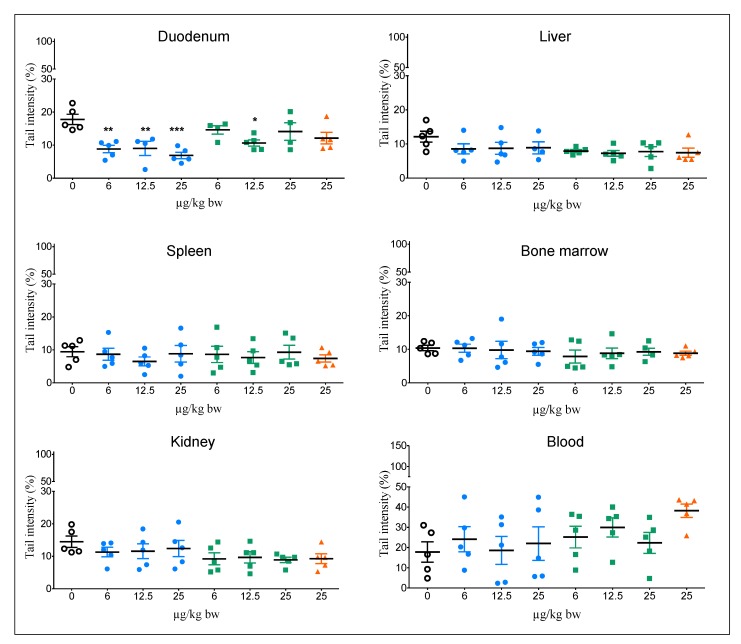
Oxidative DNA damage in different organs/tissues of rats orally exposed to Al^0^ NMs, Al_2_O_3_ NMs or AlCl_3_. A group treated with the vehicle (0, negative control) was included. Significant from control at * *p* < 0.05, ** *p* < 0.01, *** *p* < 0.001.

The data for oxidative DNA damage using the modified Fpg comet assay are shown in Figure 2. No increase in tail intensity was observed in any organ or tissue from rats treated with Al^0^ and Al_2_O_3_ NMs. However, a significant decrease in tail intensity was detected in the duodenums of rats treated with Al^0^ NMs for the three doses. An increase in oxidative lesions, although not statistically significant, was observed in the blood of rats treated with AlCl_3_ at 25 mg/kg bw.

The positive control MMS induced a significant increase in tail intensity for all organs with and without Fpg (*** *p* < 0.001).

With the exception of the positive control MMS, the number of hedgehogs was generally low for all treated groups and tissues in the modified Fpg comet assay except for kidney and spleen (Supplementary Tables S2 and S3).

### 3.2. Bone Marrow Micronucleus Assay (BMMN)

The results of the BMMN test conducted after oral exposure to Al^0^ NMs and Al_2_O_3_ NMs at 6, 12.5 or 25 mg/kg bw, and to AlCl_3_ at 25 mg/kg bw, are presented in Table 2.

The two Al-NMs did not induce any significant changes in the percentage of MN-PCEs compared to the control group. No significant myelotoxicity was found with either Al-NMs or AlCl_3_.

The positive control group treated with MMS demonstrated a significant increase in MN-PCEs and a decrease of the percentage of PCEs.

### 3.3. Micronucleus Assay in Colon

**Figure 3:**
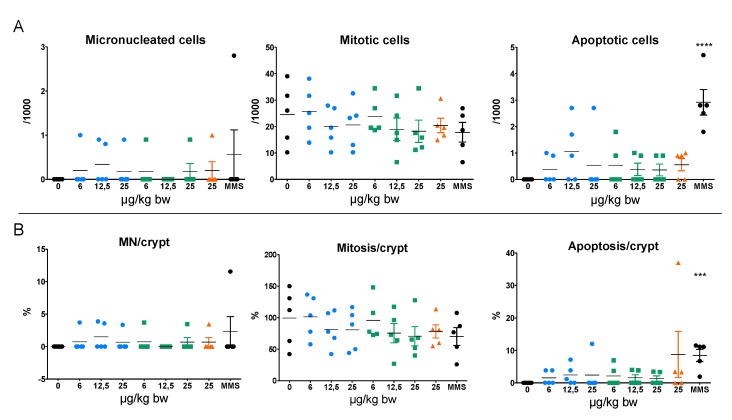

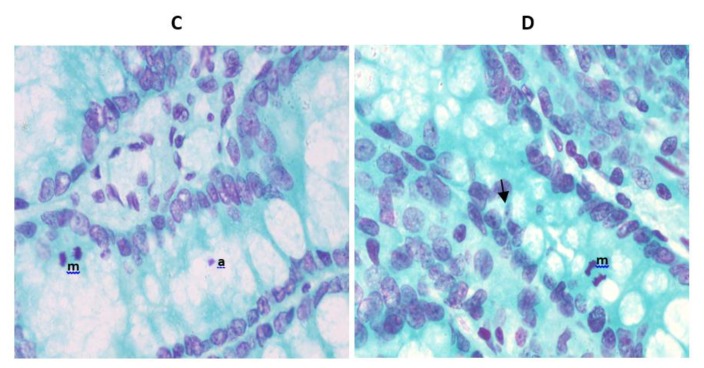
(**A**) MN, mitotic and apoptotic frequencies per 1000 cells in the colon of rats orally exposed to Al NMs and AlCl_3_ or to the solvent control (0). (**B**) MN, mitosis and apoptosis percentages per crypt. Results correspond to individual values with mean ± SD (n = 5). Significant from control at *** *p* < 0.001, **** *p* < 0.0001 compared to the vehicle control. (**C**,**D**) Schiff’s reagent and fast green counterstained colon sections of rats treated with MMS (**C**) and Al^0^ NMs at 12.5 mg/kg bw (**D**). Black arrow indicates micronuclei in cells. a: apoptosis, m: mitosis.

The micronucleus assay in colon (Figure 3) showed that the two Al-NMs and AlCl_3_ did not induce any significant increase in micronucleated cells compared to control rats. Similarly, no increase in mitosis and apoptosis was detected with the three Al forms. With the positive control MMS, only a significant increase in the level of apoptotic figures was detected.

## 4. Discussion

To date, published data on the oral genotoxicity of Al-containing NMs are scarce. In this study, we investigated the genotoxic potentials of two nanoforms (Al^0^ and Al_2_O_3_) by applying the in vivo OECD guidelines for the comet assay in several key tissues/organs [27], and the micronucleus assay on bone marrow [32] and on colons. In addition, we have compared the responses with the ionic form AlCl_3_.

The micronucleus assay both on BM and on colon was negative with Al^0^ and Al_2_O_3_ NMs up to 25 mg/kg bw indicating that no chromosome or genome mutations were induced in these two organs. Similarly, a negative response was observed in the blood of rats exposed orally to Al_2_O_3_ NMs (30 nm and 40 nm) at a unique dose of 500 mg/kg bw. However, doses above 1000 mg/kg bw induced the formation of MN, concomitantly to high levels of Al content in several organs, including blood, liver, spleen and kidney [11]. Nevertheless, such doses are very high and far from human exposure. Intraperitoneal injection of nano- and bulk-Al from 300 to 1200 µg/kg bw in male and female mice did not provoke any increase of MN in BM [33]. In vitro, Al_2_O_3_ NMs were found to induce MN in a concentration dependent manner after 24 h exposure from 0.5 µg/mL in Chinese hamster cells CHOK1 [19] and in human fibroblasts at 13.3 and 26.6 µg/cm^2^ (50 and 100 µg/mL) [16]. In contrast, no MN increase was found in human blood lymphocytes [34] or RAW 264 macrophages exposed for 72 h to Al_2_O_3_ NMs [35]. A negative response was also observed with the chromosome aberrations assay in human lymphocytes with Al_2_O_3_ NMs [18]. Similarly, with the same NMs and dispersion protocol used in our study, we did not detect any increase of MN in human intestinal Caco-2 cells and hepatic HepaRG cells for Al^0^ and Al_2_O_3_ NMs up to 80 µg/cm^2^ and for AlCl_3_ following 24 h exposure [36].

No DNA damage was observed in the alkaline comet assay in any of the six organs/tissues investigated from rats orally-exposed to Al^0^ NMs up to 25 mg/kg bw. Nevertheless, a decrease of the tail intensity was observed in duodenum that may be a result of cross-links induced by NMs, preventing the DNA migration, as described in the literature [37]. In fact, such a cross-linking effect has been recently described in plants treated with AlCl_3_, and it was suggested that Al may interact with DNA in an electrostatic manner [38]. Further data are necessary to confirm this hypothesis. Due to interference of Al^0^ NMs with the assay in vitro, the genotoxic potential of these NMs on hepatic and intestinal cell lines is unclear [36].

In this study we have shown induced DNA damage in BM of rats exposed to 25 mg/kg bw Al_2_O_3_ NMs but not in the other organs or tissues investigated. Balasubramanyam et al. (2009) observed an increase of DNA damage in blood of rats up to 48 h after a single treatment with 30 and 40 nm Al_2_O_3_ NMs by gavage; however, this was observed at very high doses (above 1000 mg/kg) [11]. Recently, DNA fragmentation due to cell death (both necrosis and apoptosis) was reported in liver and kidney of rats treated orally for 75 days with 70 mg/kg bw Al_2_O_3_ NMs [39]. Two other studies have investigated the genotoxic potential of Al_2_O_3_ NMs after intraperitoneal administration. These have demonstrated DNA damage in blood after 6 weeks of repeated exposure at 1.25 mg/kg bw [40] and in the brains of rats 48 h after a single intraperitoneal administration of Al_2_O_3_ NMs, although at very high doses (from 4 to 8.5 g/kg bw) which were evaluated as lethal (from 30 to 65% of the LD50) in the same study [41]. In this study, the DNA damage was correlated with Al accumulation in several organs, including the brain.

In vitro, time- and concentration-dependent DNA damage was found in Chinese hamster lung fibroblasts [33] and in liver HepG2 cells [17] exposed to Al_2_O_3_ NMs from 30 µg/mL. However, other studies have reported negative responses in human peripheral blood cells and human embryonic kidney cells up to 100 µg/mL following a 3 h treatment [42], and in human lymphocytes at 100 µg/mL after a 24 h exposure [18]. Recently, it was shown that Al_2_O_3_ NMs can inhibit DNA polymerase replication but without affecting mutation rate compared to controls [43].

To investigate if oxidative DNA damage can be increased with Al NMs, we performed the Fpg-modified comet assay. Neither Al^0^ nor Al_2_O_3_ NMs induced oxidative DNA damage in six different organs of rats exposed to 6–25 mg/kg bw. Although in this study we did not observe an increase in oxidative DNA damage with Al_2_O_3_ NMs, Shah et al. (2015) reported an increase in 8-oxo guanine, an oxidized DNA base, in vivo in the brain of mice treated intraperitoneally with 50 mg/kg Al_2_O_3_ NMs [44]. Similar results were observed in vitro in mouse neuronal cells treated with Al_2_O_3_ NMs from 100 to 150 µg/mL [44].

Several metal oxide NMs have been shown to induce oxidative stress [13,14,18,33,45,46] which in some cases was correlated with DNA damage [47]. Several in vivo studies with Al NMs have described a concentration-dependent increase in oxidative stress with Al_2_O_3_ NMs in several tissues including liver and kidney induced after acute (3 days) and chronic (up to 21 days) oral exposures to rats at doses ranging from 500 to 2000 mg/kg bw [13,14]. Moreover, oxidative stress was observed in several organs of rodents following acute and repeated intraperitoneal exposure [44,48]. Using doses of Al_2_O_3_ NMs more consistent with daily human exposure (0.5 to 70 mg/kg bw), oxidative stress was detected in rat liver, kidney and erythrocytes after repeated daily oral exposure [39,45,49]. In vitro, some evidence that exposure to Al_2_O_3_ NMs can induce oxidative stress has been also reported in various cell lines following 24 h exposure [18,33,44,46,47] that can be rapidly repaired depending on the concentration [47]. In contrast, Demir et al. (2015) did not observe any oxidative DNA damage in human peripheral blood cells exposed 3 h to Al_2_O_3_ NMs up to 10 µg/mL [50]. It was also shown that impairment of mineral homeostasis linked to oxidative stress could be induced by Al_2_O_3_ NMs in hippocampi of rats after intravenous injections [51].

In agreement with our results for Al^o^ and Al_2_O_3_ NMs, no increase in MN was observed in either in BM or colons of rats exposed to 25 mg/kg bw AlCl_3_. In other studies, however, AlCl_3_ was found to increase the number of MN in BM after a unique oral exposure to mice at 50 mg/kg bw [22]. Likewise, increases in MN frequency were observed in rat liver after a 30 day oral exposure [52] or with a 5 mg/kg bw daily intra-peritoneal exposure for 10 weeks [53]. Interestingly, the induction of MN formation following oral exposure could be decreased with an antioxidant treatment (propolis or borax) [52,54] suggesting that the effects of AlCl_3_ where linked to oxidative stress as described elsewhere [55,56,57]. Nevertheless, apoptosis has been described as a cause of DNA fragmentation in the liver of mice following acute exposures to AlCl_3_ (25 mg/kg by ip) [58]. Aluminum acetate (50 mg/kg) was also found to induce chromosome aberrations in BM of mice after both single and seven consecutive day intraperitoneal administration while the MN response was only positive after repeated exposure [59]. In vitro, AlCl_3_ was reported to increase the number of MN and chromosomal aberrations in peripheral blood lymphocytes after 24 h exposure between 1 and 10 µg/mL [60].

We did not observe any increase in DNA damage with the comet assay in duodenum and liver, in agreement with our in vitro results on liver and intestinal human cells [36]. No DNA damage, with or without Fpg, was detected in the other organs and tissues investigated, with the exception of blood where a slight increase of oxidative DNA damage was observed. Indeed, DNA damage in response to AlCl_3_ exposure was reported in vitro in human peripheral blood cells [20,21]. Oxidative stress in response to AlCl_3_ treatment was demonstrated to correlate with oxidative DNA damage induced in human peripheral blood cells [21] and the increase of GSH/GSSG ratio and Hsp70 mRNA levels in human neuroblastoma cells [61].

While a negative response in the MN assay was observed with the three forms of Al both in vitro and in vivo, the comet assay indicated effects of Al_2_O_3_ NMs on BM and with AlCl_3_ on blood. No correlation between the in vivo genotoxicity of Al NMs and the ionic salt AlCl_3_ was observed in our study, and we therefore cannot conclusively determine the potential effect of aluminum ions released from the Al NMs. Although we have previously reported a low solubility of these Al-containing NMs in an in vitro digestion system [25], estimating the solubility of NMs in the intestinal fluid in vivo remains challenging. Therefore, no clear conclusion can be drawn on the role, if any, of metallic ions in the genotoxic effects observed. The differences due to the route of exposure, the Al form tested, the organs studied and the dose of exposure can explain the discrepancies of the responses in the different publications.

## 5. Conclusions

Our results indicate that Al^0^ and Al_2_O_3_ NMs do not induce chromosomal mutations detected by the micronucleus assay in BM or the colons of rats exposed orally to Al-containing NMs at doses from 6 to 25 mg/kg bw. However, the comet assay showed some increase of DNA damage only in BM with Al_2_O_3_ NMs, while AlCl_3_ induced slight oxidative DNA damage in blood. No clear relationship between genotoxic effects and ion release could be determined. Challenges remain in the evaluation of the genotoxicity of Al-containing NMs, and further work is necessary in order to correlate these data with the measurement and characterization of Al NMs in the different organs and body fluids, including data on solubility in vivo.

## Figures and Tables

**Table 1:** Summary of nanomaterial (NM) characteristics.

NM	NM-Code	Average Particle Size ^a^ (nm)	SSA ^b^ (m^2^/g)	Purity ^c^	Bulk Density, True Density ^d^ (g/cm^3^)	Morphology	Pdi ^e^	Z-Average Size in the Stock Solution Dispersion at 0 h ^e^ (nm)	Solubility ^f^ (24h) (%)
**Al^0^**	NM-0015-HP	18	40–60	>99%	2.7	Spherical	0.17 ± 0.004	254 ± 4	0.48 ± 0.02
					0.008–0.2				
**γ-Al_2_o_3_**	NM-0036-HP	20	<200	99%	-	Spherical	0.23 ± 0.015	168 ± 3	0.15 ± 0.01
					0.9				

^a^ Average particle size was determined by TEM. ^b^ Average specific surface area (SSA) was determined by Brunauer-Emmet-Teller (BET). Purity was determined by X-ray powder diffraction (XRD). ^d^ Density was assessed by normal volumetric test. ^e^ Pdi and Z-average size were assessed by dynamic light scattering (DLS) using Malvern Zetasizer (Malvern Instruments, Malvern, UK) equipped with a 633-nm laser diode operating at an angle of 173°. ^f^ Ion release was performed using with a quadrupole inductively coupled plasma mass spectrometry (ICP-MS). ^a,b,c,d^ Information provided from the supplier. ^e,f^ Dispersion stock solution.

**Table 2:** Genotoxicity of Al^0^ NMs, Al_2_O_3_ NMs and AlCl_3_ in rats treated orally detected by the micronucleus assay in bone marrow.

		Genotoxicity	Myelotoxicity
		Micronucleated PCE/2 000 PCE	%PCEs
	Doses (mg/kg bw/day)	Mean ± SD	Mean ± SD
**Control**		1.3 ± 0.91	72.5 ± 26
**Al^0^**	**6**	2.0 ± 2.0	66.3 ± 13
	**12.5**	1.5 ± 0.8	71.5 ± 8
	**25**	2.1 ± 1.0	74.7 ± 16
**Al_2_O_3_**	**6**	1.1 ± 0.7	68.4 ± 18
	**12.5**	1.6 ± 1.3	65.8 ± 17
	**25**	0.9 ± 0.8	56.7 ± 17
**AlCl_3_**	**25**	1.5 ± 0.7	65.9 ± 19
**MMS**	**100, 100, 80**	16.7 ± 3.7 *	44.5 ± 12

NCE: normochromatic erythrocytes; PCEs: polychromatic erythrocytes; results correspond to mean ± SD, n = 5; * *p* < 0.001 with the Pearson X2 with Yate’s correction.

## Supplementary Materials

The following are available online at https://www.mdpi.com/2079-4991/10/2/305/s1. Figure S1: Equivalence between the doses administrated between mass and Al content., Table S1: Percentage of hedgehogs scored on the comet slides without Fpg. Mean ± SD (n = 5 animals per condition except for control duodenum n = 4)., Table S2: Percentage of hedgehogs scored on the comet slides with Fpg. Mean ± SD (n = 5 animals per condition).
