# Supplementary material for: Genotoxicity of Aluminum and Aluminum Oxide Nanomaterials in Rats Following Oral Exposure
Source: Nanomaterials (Basel). 2020 Feb 11;10(2):305. doi: 10.3390/nano10020305 (PMC7075173; doi:10.3390/nano10020305)
Supplement: Supplementary file 1 [file nanomaterials-10-00305-s001.pdf]

nanomaterials

MDPI

Supplementary

Genotoxicity of Aluminum and Aluminum Oxide Nanomaterials in Rats Following Oral Exposure

Pégah Jalili [1], Sylvie Huet [1], Rachelle Lanceleur [1], Gérard Jarry [1], Ludovic Le Hegarat [1], Fabrice Nesslany [2], Kevin Hogeveen [1] and Valérie Fessard [1,*]

[1] Unité de Toxicologie des Contaminants, Agence Nationale de Sécurité Sanitaire (ANSES), 10 B rue Claude Bourgelat, 35306 Fougères, France; pjalili@uci.edu (P.J.); sylvie.huet@anses.fr (S.H.); rachelle.lanceleur@anses.fr (R.L.); gerard.jarry@anses.fr (G.J.); ludovic.lehegarat@anses.fr (L.L.H.); kevin.hogeveen@anses.fr (K.H.)

[2] Institut Pasteur de Lille, Laboratoire de toxicologie génétique, 1 Rue du Professeur Calmette, 59019 Lille CEDEX, France; fabrice.nesslany@pasteur-lille.fr

* Correspondence: Valerie.fessard@anses.fr; Tel.: +33-(0)2-9994-6685

Received: 9 January 2020; Accepted: 2 February 2020; Published: date

Table S1. Equivalence between the doses administrated between mass and Al content.

Al form	6.25 mg/kg	12.5 mg/kg	25 mg/kg
Al^0 NMs	6.25 Al mg/kg	12.5 Al mg/kg	25 mg Al/kg
Al_2O_3 NMs	3.31 mg Al/kg	6.625 Al mg/kg	13.25 mg Al/kg
$AlCl_3$	0.69 mg Al/kg	1.375 mg Al/kg	2.75 mg Al/kg

Table S2: Percentage of hedgehogs scored on the comet slides without Fpg. Mean ± SD (n=5 animals per condition except for control duodenum n=4).

**Tissue/organ**	**Control**	**Al^0**			**Al_2O_3**			**$AlCl_3$**	**MMS**
**Dose (mg/kg)**		**3 x 6.25**	**3 x 12.5**	**3 x 25**	**3 x 6.25**	**3 x 12.5**	**3 x 25**	**3 x 25**	**2 x 100 + 1 x 80**
**Duodenum**	1.05 ± 1.09	0.0 ± 0.0	1.03 ± 1.67	0.0 ± 0.0	0.0 ± 0.0	0.0 ± 0.0	0.0 ± 0.0	2.03 ± 2.77	1.07 ± 1.68
**Liver**	1.40 ± 2.21	0.52 ± 0.72	2.58 ± 1.97	2.78 ± 3.43	1.82 ± 1.46	1.55 ± 1.68	1.29 ± 1.82	0.78 ± 1.16	0.52 ± 0.71
**Kidney**	4.19 ± 4.06	3.28 ± 3.03	6.76 ± 4.76	7.56 ± 2.92	6.71 ± 5.30	6.59 ± 6.14	3.04 ± 2.69	6.14 ± 3.65	1.04 ± 1.42
**Spleen**	1.53 ± 2.27	0.78 ± 1.16	0.26 ± 0.59	0.78 ± 1.16	2.62 ± 5.15	1.82 ± 1.46	2.26 ± 3.07	1.05 ± 1.09	6.11 ± 13.66
**Bone marrow**	0.2 ± 0.45	1.28 ± 2.19	1.80 ± 1.94	1.80 ± 2.12	4.68 ± 4.06	1.82 ± 1.46	2.74 ± 3.60	2.07 ± 1.46	5.50 ± 7.66
**Blood**	2.03 ± 2.59	1.55 ± 1.68	1.56 ± 1.40	0.26 ± 0.59	2.07 ± 1.46	0.53 ± 0.72	1.30 ± 1.57	1.82 ± 1.46	2.20 ± 4.37

nanomaterials

Table S3: Percentage of hedgehogs scored on the comet slides with Fpg. Mean ± SD (n=5 animals per condition).

Tissue/organ	Control	Al^0			Al_2O_3			$AlCl_3$	MMS
Dose (mg/kg)		3 x 6.25	3 x 12.5	3 x 25	3 x 6.25	3 x 12.5	3 x 25	3 x 25	2 x 100 + 1 x 80
**Duodenum**	2.75 ± 4.26	0.52 ± 1.16	0.26 ± 0.59	0.52 ± 0.16	0.0 ± 0.0	0.0 ± 0.0	0.0 ± 0.0	0.39 ± 0.88	100 ± 0.0
**Liver**	8.03 ± 16.49	2.14 ± 1.50	1.03 ± 1.67	1.03 ± 1.67	0.77 ± 1.72	2.26 ± 3.13	3.03 ± 2.99	2.81 ± 2.21	100 ± 0.0
**Kidney**	7.18 ± 5.01	8.61 ± 4.71	5.03 ± 1.90	6.37 ± 2.70	3.68 ± 4.36	5.46 ± 3.12	4.00 ± 3.41	5.89 ± 3.91	100 ± 0.0
**Spleen**	0.52 ± 1.16	0.80 ± 0.84	0.53 ± 0.72	0.53 ± 0.72	1.48 ± 3.31	2.05 ± 2.30	1.29 ± 1.82	0.78 ± 1.16	100 ± 0.0
**Bone marrow**	1.03 ± 1.67	1.80 ± 1.10	1.29 ± 1.82	1.30 ± 1.57	2.51 ± 3.15	2.30 ± 2.39	2.55 ± 2.38	1.80 ± 2.12	100 ± 0.0
**Blood**	0.26 ± 0.59	1.53 ± 2.27	1.53 ± 2.27	1.28 ± 2.19	2.80 ± 2.40	1.81 ± 1.73	0.26 ± 0.59	1.54 ± 2.08	100 ± 0.0
